# Literature Review and a Relevant Case of Pediatric Collagenous Gastritis: A Rare but Important Etiology of Iron-Deficiency Anemia

**DOI:** 10.1097/PG9.0000000000000351

**Published:** 2023-09-08

**Authors:** Madison Romano, Natalia Plott, Andrew Galligan, Racha Khalaf

**Affiliations:** From the * University of South Florida Morsani College of Medicine, Tampa, FL; † Department of Pediatrics, University of South Florida Health Morsani College of Medicine, Tampa, FL; ‡ Department of Pediatric Hematology and Oncology, University of South Florida Health Morsani College of Medicine, Tampa, FL; § Department of Pediatric Gastroenterology, Hepatology and Nutrition, University of South Florida Health Morsani College of Medicine, Tampa, FL.

**Keywords:** collagenous gastritis, iron deficiency anemia, celiac disease, autoimmune

## Abstract

An adolescent male with fatigue, weight loss, and iron-deficiency anemia failed to improve with iron supplementation and a gluten-free diet. Endoscopy revealed collagenous gastritis. Pediatric patients with refractory iron deficiency and family history of autoimmune disorders should be referred to pediatric gastroenterology for evaluation of collagenous gastritis and celiac disease.

## INTRODUCTION

Collagenous gastritis (CG) is a rare clinical diagnosis characterized by subepithelial patches of collagen deposits and inflammatory infiltrates in the gastric lamina propria usually limited to the corpus (1). The etiology of the disease is poorly understood, with few cases documented in the English literature since it was first reported in 1989 (2). CG may be underdiagnosed as it presents differently in pediatric versus adult populations (1,3), and its symptoms may vary greatly from patient to patient (1–3). Common symptoms include abdominal pain, nausea, anorexia, and diarrhea but other symptoms are ambiguous and may not be limited to gastrointestinal disorders, such as anemia, fatigue, or thrombocytopenia, making it difficult to diagnose patients with CG (4).

CG has been associated with autoimmune disorders. In patients with a family history of autoimmune disease, it is necessary to consider evaluating for other conditions such as celiac disease, which may have similar symptoms to CG including gastrointestinal symptoms and iron-deficiency anemia (3,4).

Herein, we report a case of pediatric CG in a patient presenting with refractory iron-deficiency anemia and nonspecific symptoms, along with a family history of autoimmune disease.

## CASE REPORT

A 16-year-old male with a history of refractory iron-deficiency anemia and asthma presented to the pediatric gastroenterology clinic with fatigue, dizziness, lightheadedness, and inability to focus, which began 2 to 3 months before his evaluation. He had an associated unintentional weight loss of 20 pounds over the prior 6 months. Family history was pertinent for a brother with a history of Hodgkin’s lymphoma and celiac disease. Initial bloodwork performed by his primary care physician was pertinent for a hemoglobin of 9.9 g/dL, hematocrit of 33.2%, mean corpuscular volume of 74.3 fl, ferritin of 5 ng/mL, total iron saturation of 5%, iron level of 19 mcg/dL, and red cell distribution width of 15.2%, consistent with iron-deficiency anemia leading to initiation of oral pharmacological therapy with ferrous sulfate 325 mg once daily. He was evaluated by pediatric hematology 1 month later, and repeat bloodwork revealed minimal improvements in hemoglobin, low total iron, and low ferritin at 35 ng/mL, despite reported adherence to oral iron supplementation in the setting of ongoing symptoms. Total iron saturation did improve to 34%. The patient’s iron supplementation was increased to twice daily, but there was still no improvement in symptoms at 1 month follow-up.

**FIGURE 1.**
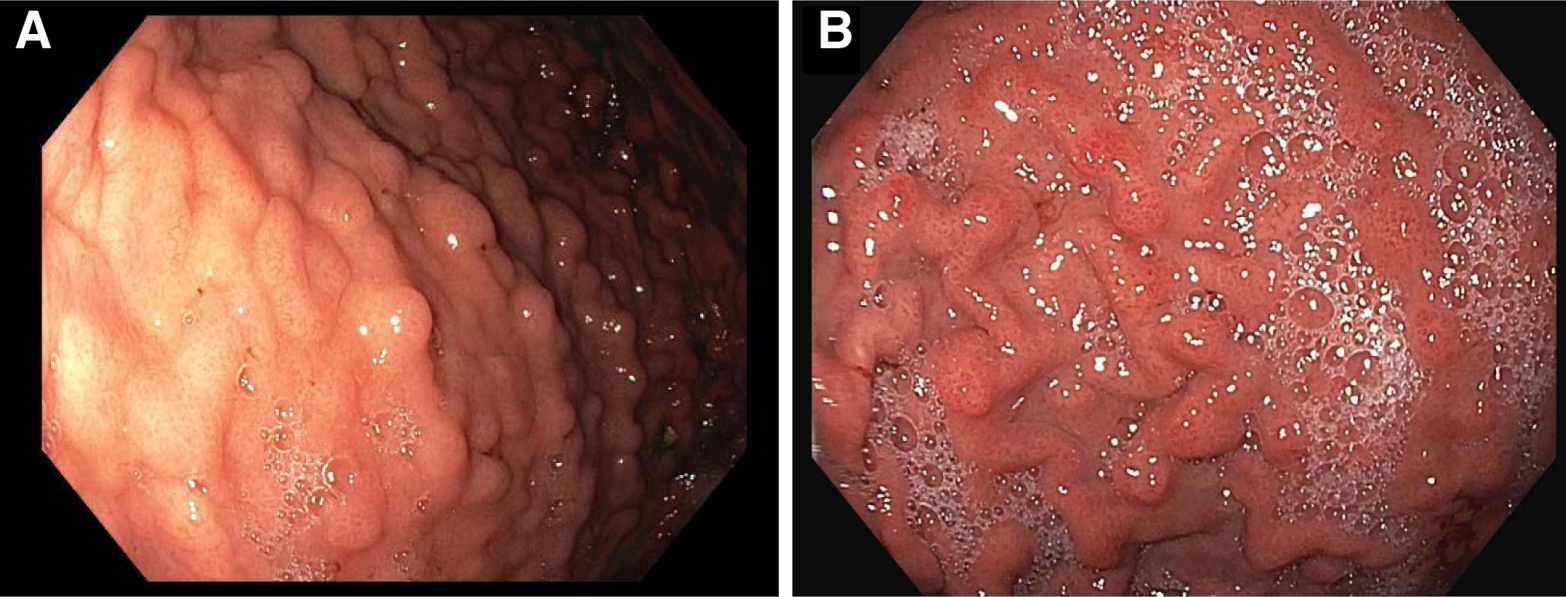
A) Nodularity of the gastric corpus, a characteristic endoscopic finding in collagenous gastritis. Within the nodular lesions, the depressed mucosal pattern is a result of inflammation and collagen deposition, leading to an atrophic appearance. B) Follow-up endoscopy at 18 months postdiagnosis revealed that the gastric body had patchy erythema and visually improved nodularity.

**FIGURE 2.**
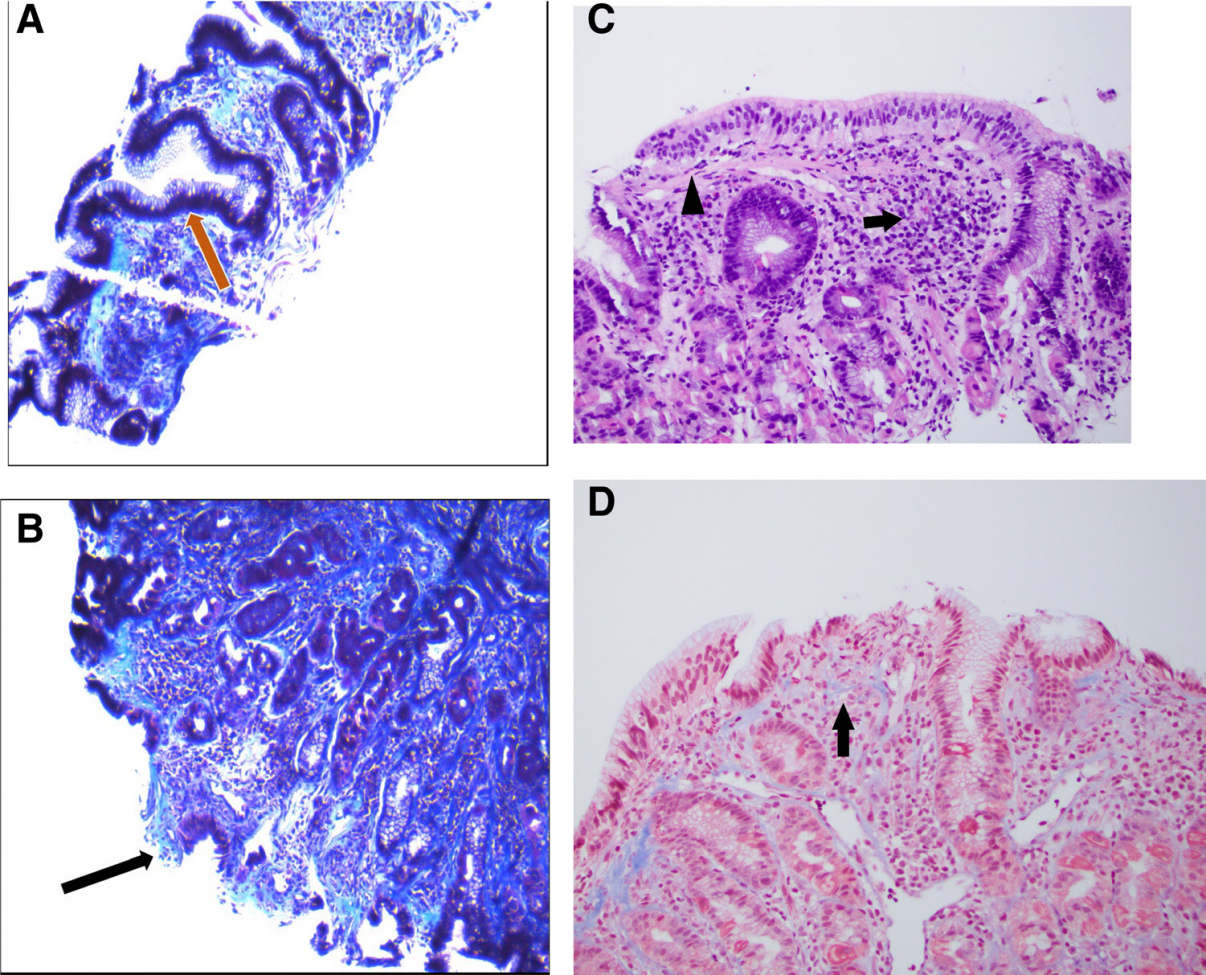
Trichrome stained gastric biopsies of antral and fundic mucosa with increased lymphoplasmacytic cells within the lamina propria and lymphoid aggregates with reactive germinal centers. A) Dark blue subepithelial thick collagen bands noted within the lamina propria (red arrow) along with (B) areas of superficial epithelial erosions (black arrow). C) Antrum (20×): The histologic sections demonstrate antral type gastric mucosa with a lymphoplasmacytic infiltrate in the lamina propria (arrow) without neutrophils in the epithelium, consistent with chronic inactive gastritis. The basement membrane is not thickened (arrowhead). D) Antrum, trichrome stain (20×): A trichrome stain demonstrates normal whispy staining of the subepithelial collagen, which is not increased.

The patient was then referred to pediatric gastroenterology to determine if there was a luminal etiology for poor iron absorption. The patient denied abdominal pain, nausea, or vomiting. Additionally, he reported that he had been following a gluten-free diet for months since his brother’s diagnosis of celiac disease and before the onset of his symptoms; the patient had never undergone human leukocyte antigen (HLA) genetic testing for celiac disease. The patient reported stooling 3 to 4 times per day. Stools were not melenic nor did they contain any bright red blood. The differential for his presentation included celiac disease, eosinophilic gastritis, lymphocytic gastritis, autoimmune gastritis, inflammatory bowel disease, and *Helicobacter pylori* gastritis. A stool evaluation was obtained including a hemoccult and a fecal calprotectin, both of which were negative. A celiac screen was notable for a positive tissue transglutaminase (TTG) immunoglobulin A (IgA) of 4 U/mL (normal <4 U/mL), despite the patient being on a gluten-free diet. The patient was instructed to resume a gluten-containing diet for 2 weeks before esophagogastroduodenoscopy. Visually, the endoscopy revealed a significantly nodular gastric body (Fig. 1) but otherwise a grossly normal esophagus and duodenum. Histology revealed thickened subepithelial collagen bands, greater than 15 mm, wrapped around subsurface capillaries (Fig. 2). The visual and histological findings were consistent with a diagnosis of CG. No evidence of celiac disease was noted within histology despite the endoscopist obtaining 6 duodenal samples.

Other etiologies for a false positive TTG were considered and included: autoimmune hepatitis, which was unlikely due to normal liver enzymes; viral gastroenteritis; and inflammatory bowel disease. It is possible that the TTG was positive but down-trending secondary to adherence to a gluten-free diet. However, the family deferred HLA testing and opted to continue a gluten-free diet. Ultimately, the biopsy samples, endoscopic findings symptomology, and response to treatment were diagnostic of CG.

The patient experienced prompt improvement of his symptoms with the initiation of a prescribed proton pump inhibitor and oral iron supplementation, in conjunction with the resumption of a gluten-free diet which he elected to do, as his family follows this diet for his brother with celiac disease. His lab values normalized, with his hemoglobin increasing to 14.2, ferritin increasing to 28 ng/mL, total iron saturation increasing to 28%, and iron levels increasing to 82 mcg/dL. Repeat endoscopy at 2 months showed visual persistence of the gastric nodularity with reduced collagen thickening in biopsied sections. Colonoscopy performed at that time was visually and histologically normal.

## DISCUSSION

CG can have an insidious and vague presentation that may mimic other disease etiologies such as celiac disease. We discussed the case of an adolescent male who was diagnosed with CG presenting with nongastrointestinal symptoms, positive celiac antibodies, and iron-deficiency anemia in the setting of family history of autoimmune disease. Endoscopic and histologic evaluation for celiac disease was negative and initial titers were likely false positive, albeit disease can be patchy. Iron deficiency was secondary to CG, confirmed histologically.

Presentation for CG can vary among patients. As demonstrated by the case above, patients with CG may not present primarily with gastrointestinal symptoms despite having abnormal endoscopic findings, such as nodularity, bleeding, or ulceration (4). Prevalence of anemia and abdominal pain varies between studies; Hijaz et al. found 71% of patients presented with anemia, and only 41% presented with abdominal pain, whereas Beinvogl et al. found 78% of patients presented with abdominal pain (4,5).

Since patient presentation can be variable, it is important to obtain a thorough history to determine if CG could be on the differential.

CG has been associated with a higher incidence of autoimmune diseases. Our patient described above had a family history of celiac disease and a falsely elevated TTG IgA level. Other studies have found similar patterns in their pediatric patients with CG. In a longitudinal study by Käppi et al. (3) published in 2020, patients with CG had greater predisposition to inheriting an autoimmune disease. In a study of 15 patients, 47% of patients had a relative with an autoimmune disorder such as celiac disease, ulcerative colitis, hypothyroidism, psoriasis, and type 1 diabetes; 40% tested positive for autoantibodies, including antinuclear antibodies, smooth muscle antibodies, antithyroid peroxidase, atypical perinuclear anti-neutrophil cytoplasmic antibodies, and TTG IgA; and 53% of patients were carriers of the HLA DQ2/DQ8 (3). In another case series by Arnason et al. (6) published in 2014, of 24 pediatric CG patients, 2 had associated celiac sprue, 1 had a positive TTG test with normal histology, and 2 had associated collagenous sprue and collagenous colitis.

While the presentation of CG may be vague, the history of autoimmune disease or family history of autoimmune disease may increase the likelihood of CG. Pediatric gastroenterologists who encounter patients with abdominal symptoms and/or iron-deficiency anemia should consider evaluating the patient’s past medical history and family history to determine if there may be autoimmune disorders that could increase the likelihood of CG.

A subset of pediatric patients with CG will also have collagenous colitis, a concurrent illness which may present at times with diarrhea and hematochezia. Hijaz et al. (5) suggest that all patients with CG undergo a colonoscopy to determine if there is concurrent collagenous colitis. As there is insufficient data to recommend universal evaluation via colonoscopy, pediatric gastroenterologists should be aware that patients with CG are at an increased risk of collagenous colitis and should obtain an appropriate history to assess if further evaluation is warranted. Treatment for collagenous colitis may vary from CG and can include the use of corticosteroids.

There is no current proven treatment regimen for CG, as it can be complex and multifaceted for each patient. For our patient, iron supplementation alone was insufficient, but a combination therapy of proton pump inhibitor and iron supplementation led to significant improvement in symptomatology. Our patient had been on a gluten-free diet before his diagnosis and decided to continue this diet.

In the case series reported by Hijaz et al., treatment modalities varied from patient to patient. Of 24 patients, 13 were treated with antisecretory medications (either proton pump inhibitor or histamine-2 blockers), 9 were trialed with systemic or oral steroids, 3 received triple therapy for *Helicobacter pylori,* and other patients trialed misoprostol, furazolidone, metronidazole, and bismuth subsalicylate. A few patients in this case study also trialed therapeutic diet changes, such as a hypoallergenic diet, gluten-free diet, and parenteral nutrition. In this study, they could not find a consistent improvement in clinical outcomes despite treatment for CG. One of the patients in this study, who presented similarly to our patient with iron-deficiency anemia and thrombocytosis, received omeprazole and oral iron supplementation, which reduced the collagen bands and significantly reduced his symptoms. However, another patient with iron-deficiency anemia, abdominal pain, and pallor, who was treated with the same regimen, did not have improvements in symptoms or histopathologic findings; the addition of budesonide ultimately led to improvements in symptoms and pathological findings. Despite similar presentations and histopathology, different treatments were needed to achieve improvement in symptomology.

Case studies reported by Kamimura et al. concur with the treatment findings in Hijaz et al.; different combinations of treatments may reduce symptoms, including a regimen of proton pump inhibitors, iron supplementation, budesonide, and sucralfate (3).

In the cohort study by Beinvogl et al. published in 2021, patients were treated with oral iron supplementation and a wide range of medications including acid blockers and steroids. They found that patients with anemia responded well to oral iron supplementation, but there was no clear indication that any one intervention is helpful (4). In fact, they found most patients had resolution of their symptoms, despite retaining the same histopathology.

Choung et al. found that topically targeted budesonide improved both the symptoms and biopsy findings of CG, similar to the findings of Beinvogl et al. and Kamimura et al. In a mix of adult and pediatric patients with CG, treatment with topically targeted budesonide yielded significant clinical response, with 42% obtaining complete response and 46% achieving partial improvement; additionally, 88% of patients treated had histological improvement, 53% of whom achieved complete response and 33% achieving partial response (7).

One case report describing a 16-year-old female with both CG and collagenous colitis had treatment success with Mesalamine therapy, evidenced by decrease in symptoms and down-trending calprotectin levels (8). This therapy had not been used in other pediatric patients with CG, and it can be a consideration for CG patients who don’t respond to steroids alone.

The literature review suggests treatment for CG can be complex and must be individualized, as treatment response is patient dependent and can be unpredictable; however, it appears that proton pump inhibitors, oral iron supplementation, and anti-inflammatory medications may be beneficial for treating CG.

## CONCLUSION

Providers should consider CG on their differential for pediatric patients presenting with refractory iron-deficiency anemia, especially when there is a family history of autoimmune disorders. Symptoms of CG may mimic the presentation of hematological or gastrointestinal diseases, particularly celiac disease.

Iron-deficient CG patients may need a proton pump inhibitor or other medications in addition to iron supplementation to properly treat and reduce symptoms. This case demonstrates the importance of verifying serological testing with endoscopy, the gold standard for diagnosis of celiac disease. Pediatric gastroenterologists can work with primary care providers and pediatric hematology providers to optimize health outcomes for pediatric patients with CG.

## ACKNOWLEDGMENTS

Informed consent for this case study was obtained from the parents of the patient.
